# Open surgical repair of a loop-forming extracranial internal carotid artery aneurysm: a case report and literature review

**DOI:** 10.1590/1677-5449.202501182

**Published:** 2026-05-18

**Authors:** Cigdem Tel Ustunisik, Omer Faruk Soydas, Lara Yagci, Berk Arapi, Ozan Onur Balkanay, Deniz Göksedef, Suat Nail Omeroglu

**Affiliations:** 1 Istanbul University Cerrahpasa, Faculty of Medicine, Istanbul, Turkey.

**Keywords:** extracranial carotid artery aneurysm, open surgery, aneurysmectomy, vascular reconstruction, case report, aneurisma da artéria carótida extracraniana, cirurgia aberta, aneurismectomia, reconstrução vascular, relato de caso

## Abstract

Extracranial carotid artery aneurysms (ECCA) are rare vascular lesions that may be asymptomatic or present with compression-related symptoms, depending on their size and location. Etiologies include trauma, congenital anomalies, inflammation, atherosclerosis, or prior interventions. Major complications involve cranial nerve compression, rupture, and cerebral thromboembolism. Diagnostic and therapeutic management can be challenging, with treatment options including conservative follow-up, endovascular intervention, or open surgery. We present the case of a 76-year-old woman admitted with visual disturbances and a palpable mass on the left side of her neck. Radiological imaging revealed a 2 cm aneurysm with loop formation in the left internal carotid artery. The aneurysm was treated with open surgical aneurysmectomy and end-to-end anastomosis without grafting. Postoperative recovery was uneventful. Open surgical repair remains a safe and effective treatment, particularly in anatomically complex cases in which endovascular techniques are not feasible due to arterial tortuosity.

## Resumo

Os aneurismas da artéria carótida extracraniana são lesões vasculares raras que podem ser assintomáticas ou apresentar sintomas relacionados à compressão, dependendo do tamanho e da localização. As etiologias incluem trauma, anomalias congênitas, inflamação, aterosclerose ou intervenções prévias. As principais complicações envolvem compressão de nervos cranianos, ruptura e tromboembolismo cerebral. O diagnóstico e o manejo terapêutico podem ser desafiadores, e as opções de tratamento incluem acompanhamento conservador, intervenção endovascular ou cirurgia aberta. Apresentamos o caso de uma mulher de 76 anos, admitida com distúrbios visuais e massa palpável no lado esquerdo do pescoço. Os exames de imagem revelaram um aneurisma de 2 cm com formação em alça na artéria carótida interna esquerda. O aneurisma foi tratado por meio de aneurismectomia aberta com anastomose terminoterminal, sem uso de enxerto. A recuperação pós-operatória transcorreu sem intercorrências. A cirurgia aberta continua sendo uma opção segura e eficaz, especialmente em casos com anatomia complexa, nos quais as técnicas endovasculares não são viáveis devido à tortuosidade arterial.

## INTRODUCTION

Extracranial carotid artery aneurysms (ECCA) are a rare and life-threatening vascular pathology with an incidence of less than 1% of all peripheral arterial aneurysms.^1^ Their etiology may involve atherosclerosis, connective tissue disorders such as fibromuscular dysplasia, trauma, inflammation, infection, dissection, or prior surgeries and interventions.^2-5^ However, congenital extracranial carotid aneurysms have rarely been reported.^6^ While there may be cases that are completely asymptomatic, according to current literature reviews, 52 to 87% of cases are symptomatic.^7,8^ A pulsatile mass in the neck is the most common presentation, but symptoms may change depending on the location and size.^9^ As the aneurysm enlarges, cranial and peripheral nerve symptoms and local pharyngeal and esophageal compression symptoms may occur.^3,10^ Thromboembolic cerebral ischemia and rupture are the major complications.^1^

There is a lack of consensus regarding treatment modalities for these patients; however, conservative treatment, endovascular interventions, and open surgery are all viable options.^8,11^ Herein, we report a rare case of a saccular aneurysm of the internal carotid artery that was successfully treated with open surgery. Although endovascular techniques have gained popularity in recent years, we would like to draw attention to the fact that open surgical procedures remain safe and effective, particularly for aneurysms with complex anatomy.

This manuscript is in compliance with the Helsinki Declaration and with institutional ethical guidelines. Informed consent was obtained from the patient for publication of this case report.

## CASE REPORT

**Figure 1:**
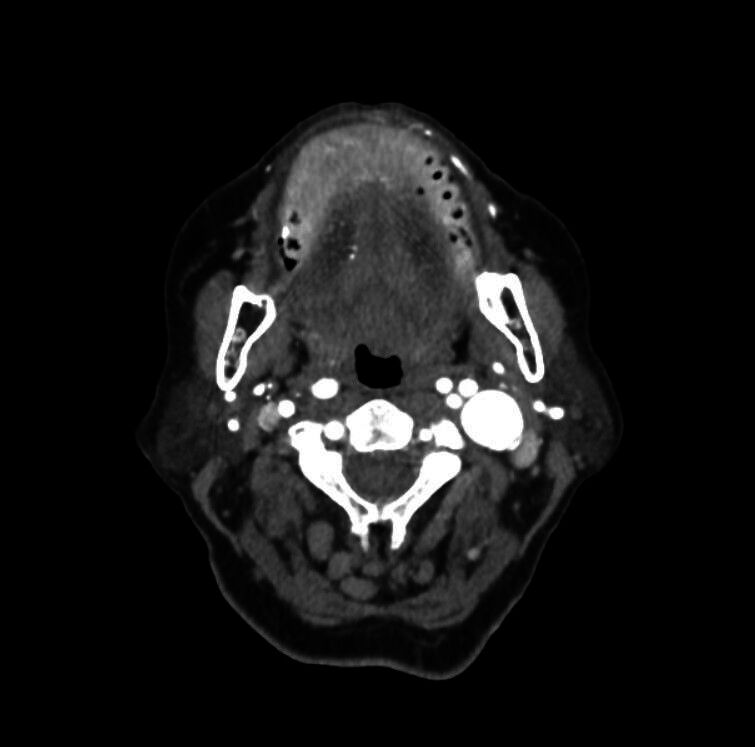
CTA showing a 20 × 20 mm tortuous aneurysm in the mid-left cervical ICA, extending towards the skull base. CTA: computed tomography angiography; ICA: internal carotid artery

A 76-year-old woman presented to an ophthalmologist with blurred vision. She was diagnosed with strabismus due to 6th cranial nerve palsy, and physical examination revealed a mass on the left side of her neck. Her medical history included coronary artery disease, type 2 diabetes mellitus, and hypertension, with no prior interventions in the neck area. Cranial magnetic resonance imaging (MRI) showed no acute pathology, only chronic age-related changes in the brain parenchyma. Craniocervical computed tomography angiography (CTA) revealed a posterolateral oriented 20 × 20 mm tortuous aneurysmal dilatation in the middle part of the left cervical internal carotid artery (ICA) extending towards the base of the skull (Figure 1).

**Figure 2:**
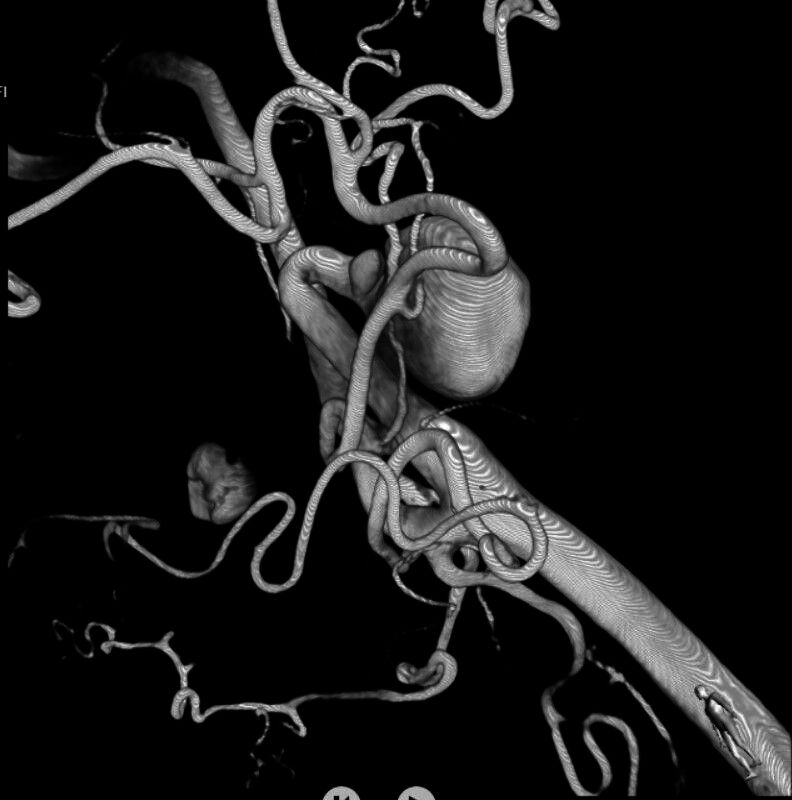
4D DSA showing noncritical stenosis in the proximal ICA, likely due to fibromuscular dysplasia, along with a 21 × 18 mm aneurysm and loop formation in the mid-cervical segment. DSA: digital subtraction angiography; ICA: internal carotid artery

Four-dimensional digital subtraction angiography (DSA) was conducted to demonstrate the nature of the disease, revealing noncritical stenosis of the proximal segment, possibly related to fibromuscular dysplasia, aneurysmatic dilatations of approximately 21 × 18 mm, and loop formation in the left mid-cervical segment of the ICA (Figure 2). The contralateral (right) ICA was patent and free of any aneurysmal or stenotic changes.

**Figure 3:**
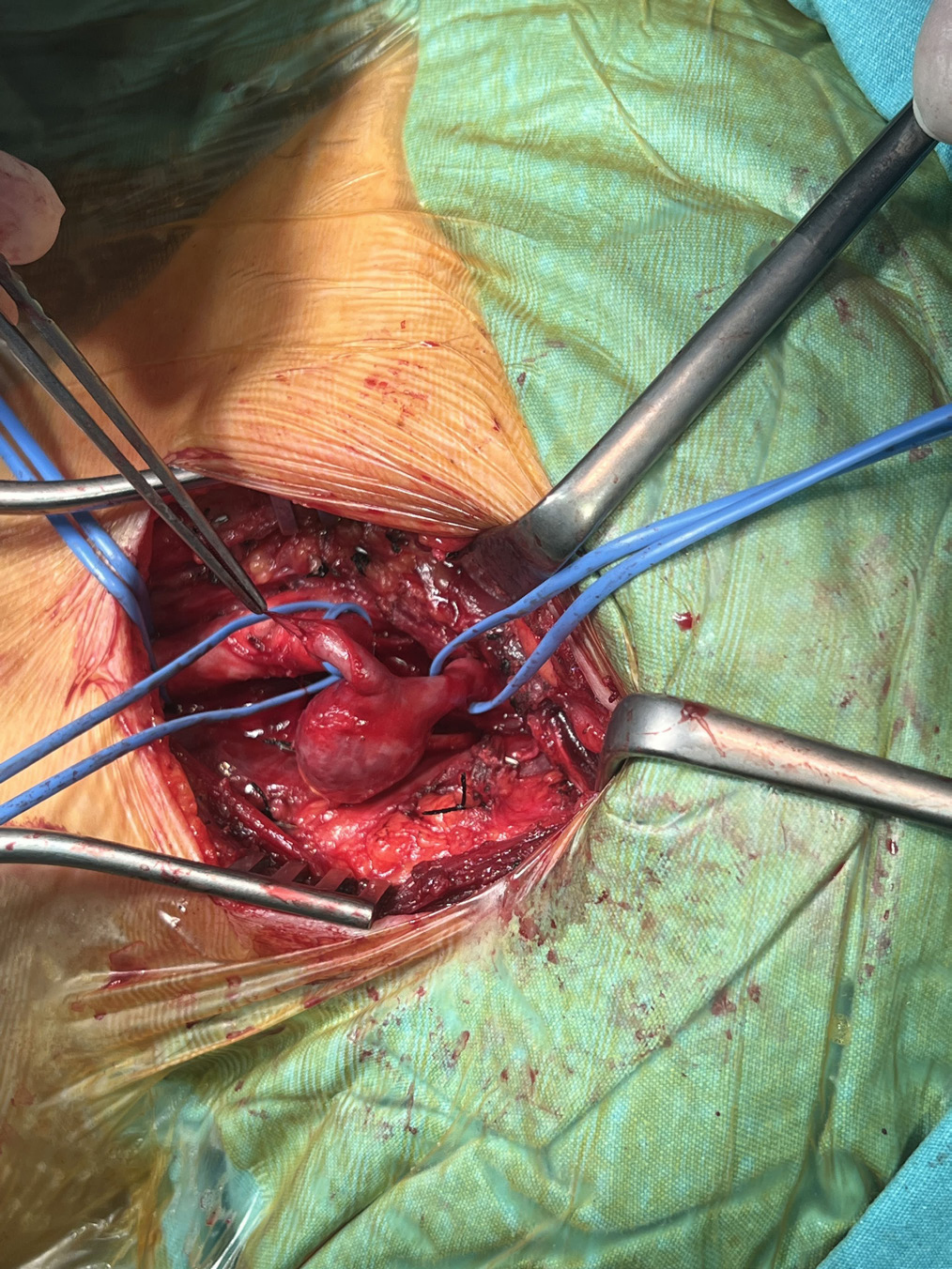
The aneurysmal sac and the proximal and distal segments of the internal carotid artery.

**Figure 4:**
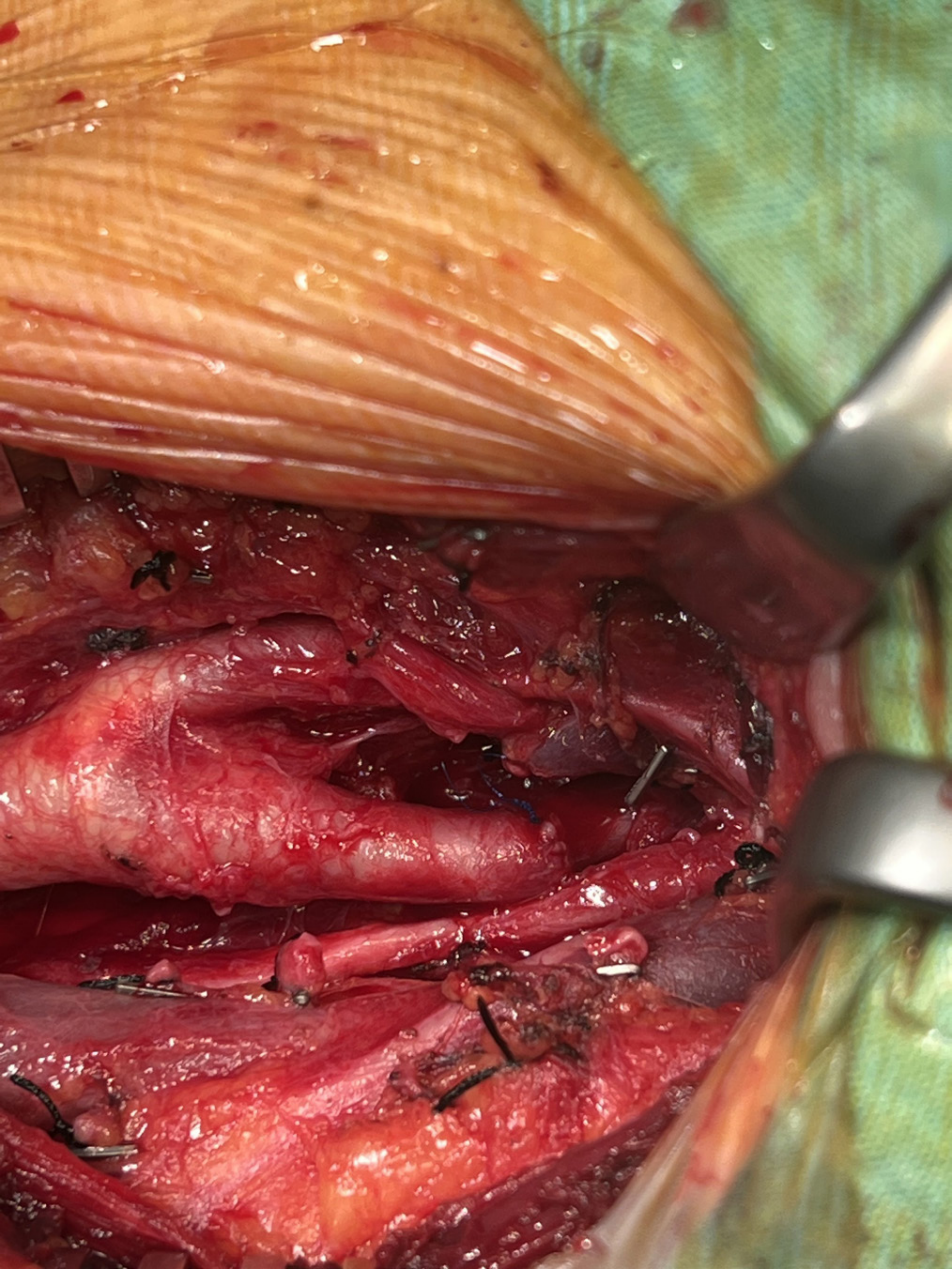
End to end anastomosis after aneurysmectomy.

The operation was performed under general anesthesia. The aneurysmal sac and the proximal and distal segments of the internal carotid artery were carefully dissected and exposed (Figure 3). After aneurysmectomy and excision of the tortuous segment of the ICA, the remaining length was long enough to perform end-to-end anastomosis, without use of a graft (Figure 4). Cerebral oxygenation was continuously monitored using near-infrared spectroscopy (NIRS), which has been shown to reliably detect intraoperative cerebral hypoperfusion during carotid clamping.^12^ As continuous NIRS monitoring showed stable bilateral cerebral oxygenation and contralateral carotid flow was normal, shunt application was not used. Preoperative angiography also confirmed the patency of the anterior communicating artery, providing additional assurance of cerebral perfusion safety during clamping. The internal carotid artery remained clamped for 10 minutes. No complications were observed in the postoperative period and the patient was discharged on the third postoperative day. Follow-up color Doppler ultrasound of the carotid arteries revealed a bilateral CCA intima-media thickness of 0.9 mm. No hemodynamic changes, aneurysmal formations, or significant stenosis were observed in the common carotid, internal carotid, or external carotid arteries and no stenotic flow patterns were detected.

## DISCUSSION

Extracranial carotid artery aneurysms (ECCA) are rare vascular lesions with diverse clinical presentations and complex anatomical characteristics, making the choice of treatment particularly challenging. Treatment strategies should be individualized, taking into account aneurysm morphology, size, location, tortuosity, symptom severity, and proximity to cranial nerves. The literature provides insights into the varied approaches for managing extracranial carotid artery aneurysms (ECCA), each with its own set of advantages and limitations. Open surgical repair is generally preferred in cases with symptomatic, large, tortuous, or true aneurysms, particularly when endovascular access is technically difficult or risky due to anatomical constraints such as loop formation.^7,13^ Although there is no universally accepted size threshold for intervention, several reports suggest that aneurysms larger than 15–20 mm have an increased risk of rupture and thromboembolic events, supporting surgical management.^14,15^ Surgical repair also allows anatomical correction and histopathological assessment, as highlighted by Argenta and Braun, who reported successful reconstruction of an extracranial carotid aneurysm with venous patch repair.^16^ This method, while effective in many cases, is associated with a higher risk of complications. However, endovascular techniques offer several advantages, including significantly reduced blood loss, shorter hospitalization, and decreased length of stay in the intensive care unit compared to traditional surgical methods.^17^ In certain complex cases, hybrid operations combining both approaches may also be considered.

Chen et al. reported that in a series involving 35 patients with extracranial carotid artery aneurysms, early complications were significantly more common in the open surgery group (43.8%) compared to the endovascular group, where no early complications were noted (P = 0.004). Their case series also demonstrated a preference for open surgery for true aneurysms, while endovascular techniques were favored for false aneurysms.^18^ Existing studies indicate that endovascular methods may be a feasible and potentially safe option for the short-term management of extracranial internal carotid artery pseudoaneurysms, despite the rarity of this condition.^19^ In distal internal carotid artery aneurysms, bare metal stents have proven to be an effective alternative to surgical resection, demonstrating full patency and complete aneurysm sac thrombosis in all patients, with no reported deaths or strokes within 30 days post-intervention, and most patients maintained successful outcomes for over 5 years.^20^ In a recent systematic review, subgroup analysis comparing covered and bare metal stents showed that while procedural success rates were similar, covered stents were associated with lower rates of reintervention, late complications, and stent-graft stenosis, alongside higher rates of aneurysm sac thrombosis.^21^ Recent studies also indicate that flow-diverting stents (FDS) are increasingly used in ECAA management, offering high rates of aneurysm occlusion and symptom resolution in suitable cases.^22^ However, use of FDS or covered stents requires a relatively straight vascular course and adequate landing zones, which can be technically challenging in tortuous or looped internal carotid segments. Therefore, device selection should be individualized based on anatomical characteristics and procedural accessibility.

Studies highlight a generally positive safety profile for endovascular procedures. In contrast, traditional surgical approaches are associated with a small incidence of transient neurological issues and other complications, which can resolve without lasting effects.^23^ Several studies have also demonstrated the safety and effectiveness of surgical treatment for extracranial internal carotid artery (EICA) aneurysms. In a large surgical series involving 64 carotid reconstructions in 57 patients, the 30-day mortality rate was 0%, and the perioperative stroke rate was only 1.6%, with an impressive 87% stroke-free rate maintained over 20 years.^11^ Similarly, Rosset et al. reported no 30-day mortality despite performing all procedures without intraluminal shunting, with only one transient stroke and two transient ischemic attacks, one of which was attributed to a clamping time exceeding 60 minutes. Follow-up imaging confirmed successful vascular reconstruction, even in patients with prior neurological symptoms.^24^ Another series of 14 surgically treated patients reported no need for reintervention and stable patency throughout the follow-up period, further supporting the long-term durability of surgical repair.^25^

Cerebral monitoring during carotid clamping plays a crucial role in preventing ischemic complications. NIRS and transcranial Doppler (TCD) are established techniques for intraoperative cerebral monitoring during carotid clamping. NIRS provides continuous, noninvasive assessment of cerebral oxygenation and has been validated against TCD for detecting hypoperfusion during carotid clamping, making it a valuable complement to other monitoring modalities.^12,26^ Despite the growing use of cerebral monitoring modalities during carotid surgery, there is still no consensus on the optimal intraoperative neuromonitoring technique. Recent reviews indicate that while NIRS is a practical, noninvasive, and cost-effective method for detecting cerebral ischemia, its positive predictive value remains limited, and the critical rSO_2_ threshold for intervention varies among devices.^27^

Preoperative assessment of intracranial circulation is also essential to predict the adequacy of collateral flow during internal carotid clamping. During internal carotid artery clamping, collateral perfusion predominantly depends on the anterior portion of the Circle of Willis, particularly through the contralateral A1 segment and the anterior communicating artery, which constitute the most favorable configuration for maintaining cerebral flow.^28^ In our patient, NIRS monitoring remained stable throughout the procedure and preoperative angiography had confirmed the patency of the anterior communicating artery, ensuring safe unilateral carotid clamping without the use of shunt application.

The potential risks of conservative management or delayed treatment are also notable. One study on surgical treatment of ECCA aneurysms reported an overall mortality and major stroke rate of 9%, with a 6% incidence of cranial nerve injury. This contrasts with non-surgical management, which can have incidence rates up to 21%. The risk of cerebral ischemia and aneurysm rupture, combined with the favorable long-term outcomes achieved through surgical intervention, highlight the advantages of a surgical approach to managing these aneurysms.^1^

Endovascular techniques have gained popularity, particularly in the management of distal extracranial carotid artery aneurysms, due to their minimally invasive nature, reduced blood loss, shorter hospital stays, and favorable early complication rates.^23^ In select cases, especially those involving non-tortuous anatomy, these methods have demonstrated successful outcomes with minimal morbidity. However, their long-term durability remains less certain, particularly in complex anatomical settings. In our patient, given the significant tortuosity and loop formation of the internal carotid artery, delivery and optimal deployment of a stent would likely have been technically challenging, potentially increasing the risk of incomplete apposition, device migration, or thromboembolic events. In contrast, open surgical repair remains the mainstay of treatment for proximally located or anatomically complex aneurysms. Techniques such as direct end-to-end anastomosis, patch angioplasty, or interposition grafting allow for definitive resection of the aneurysmal segment and precise vascular reconstruction. In experienced hands, surgical outcomes have been shown to be highly effective, with low complication rates and excellent long-term patency.

Our case exemplifies successful surgical intervention for treatment of an EICA aneurysm. We observed that the aneurysm originated from an internal carotid artery forming a 360-degree loop that significantly increases the risk of endovascular complications, including stent malposition, fracture, or occlusion. While endovascular techniques may offer less invasive options, surgery remains a critical approach for anatomically complex aneurysms, ensuring both immediate and durable results. We preferred open surgery over an endovascular approach because of the marked tortuosity, the feasibility of removing the pathological segment, and the possibility of performing a tension-free end-to-end anastomosis. The patient did not experience any complications over a two-year follow-up, further supporting the long-term effectiveness and safety of surgical intervention.

Given the rarity of extracranial internal carotid artery aneurysms, there is currently a lack of comprehensive studies comparing the superiority of endovascular versus surgical treatment. Nevertheless, existing evidence and clinical experience suggest that open surgery continues to offer a reliable and definitive solution, particularly in patients with high-risk anatomical features. While endovascular options offer important alternatives, open surgical intervention remains indispensable for ensuring long-term vascular integrity in challenging cases. Further research is essential to refine treatment algorithms and improve outcomes for this uncommon vascular condition.

## Data Availability

Data sharing does not apply to this article, as no data were generated or analyzed.

## References

[1] El-Sabrout R, Cooley DA (2000). Extracranial carotid artery aneurysms: Texas Heart Institute experience. J Vasc Surg.

[2] Garg K, Rockman CB, Lee V (2012). Presentation and management of carotid artery aneurysms and pseudoaneurysms. J Vasc Surg.

[3] Longo GM, Kibbe MR (2005). Aneurysms of the carotid artery. Semin Vasc Surg.

[4] Faggioli GL, Freyrie A, Stella A (1996). Extracranial internal carotid artery aneurysms: results of a surgical series with long-term follow-up. J Vasc Surg.

[5] Robaldo A, Persi F, Trucco A, Apostolou D (2021). Atherosclerotic saccular aneurysm of the extracranial internal carotid artery: surgical repair. Ann Med Surg (Lond).

[6] Pillai A, Arora P, Asokan R, Joseph N, Silpa S (2022). Extracranial internal carotid aneurysm manifesting with tinnitus: a rare presentation. Cureus.

[7] Fankhauser GT, Stone WM, Fowl RJ (2015). Surgical and medical management of extracranial carotid artery aneurysms. J Vasc Surg.

[8] Welleweerd JC, den Ruijter HM, Nelissen BG (2015). Management of extracranial carotid artery aneurysm. Eur J Vasc Endovasc Surg.

[9] Nordanstig J, Gelin J, Jensen N, Osterberg K, Strömberg S (2014). National experience with extracranial carotid artery aneurysms: epidemiology, surgical treatment strategy, and treatment outcome. Ann Vasc Surg.

[10] Aydin F, Ogul H, Kantarci M (2025). Unusual manifestation of an extracranial carotid artery aneurysm: airway compression. Ear Nose Throat J.

[11] Attigah N, Külkens S, Zausig N (2009). Surgical therapy of extracranial carotid artery aneurysms: long-term results over a 24-year period. Eur J Vasc Endovasc Surg.

[12] Wang Y, Li L, Wang T (2019). The efficacy of near-infrared spectroscopy monitoring in carotid endarterectomy: a prospective, single-center, observational study. Cell Transplant.

[13] Mukherjee P, Huilgol R, Graham A, Fagan P (2016). Open and endovascular repair of aneurysms affecting the distal extracranial internal carotid artery: case series. J Laryngol Rhinol Otol.

[14] Choi E, Gwon JG, Kwon SU, Lee DH, Kwon TW, Cho YP (2022). Management strategy for extracranial carotid artery aneurysms: a single-center experience. Medicine (Baltimore).

[15] AXiEr A, Turhon M, Maimaiti A (2024). Management of large or giant extracranial carotid artery aneurysms: a single-center experience. BMC Neurol.

[16] Argenta R, Braun SK (2015). Surgical repair of an extracranial carotid aneurysm. J Vasc Bras.

[17] Qiu J, Zhou W, Zhu X (2020). Treatment of extracranial carotid artery aneurysm: fifteen years’ experience at a single institution. Ann Vasc Surg.

[18] Chen Z, Chen L, Zhang J (2019). Management of extracranial carotid artery aneurysms: a 6-year case series. Med Sci Monit.

[19] Gupta R, Thomas AJ, Masih A, Horowitz MB (2008). Treatment of extracranial carotid artery pseudoaneurysms with stent grafts: case series. J Neuroimaging.

[20] Welleweerd JC, de Borst GJ, de Groot D, van Herwaarden JA, Lo RT, Moll FL (2015). Bare metal stents for treatment of extracranial internal carotid artery aneurysms: long-term results. J Endovasc Ther.

[21] Li Z, Chang G, Yao C (2011). Endovascular stenting of extracranial carotid artery aneurysm: a systematic review. Eur J Vasc Endovasc Surg.

[22] Cinar C, Akgul E, Elek A (2025). Flow-diverting stents in the management of extracranial carotid artery aneurysms. Diagn Interv Radiol.

[23] Angiletta D, Pulli R, Marinazzo D, Frotino P, Maiellaro L, Regina G (2014). Surgical and endovascular treatment of extracranial carotid artery aneurysms: early and long-term results of a single center. Ann Vasc Surg.

[24] Rosset E, Albertini JN, Magnan PE, Ede B, Thomassin JM, Branchereau A (2000). Surgical treatment of extracranial internal carotid artery aneurysms. J Vasc Surg.

[25] Yildiz Z, Kaygin MA, Ozkara T (2022). Extracranial carotid artery aneurysm and surgical treatment. Turk J Vasc Surg..

[26] Yun WS (2017). Cerebral monitoring during carotid endarterectomy by transcranial Doppler ultrasonography. Ann Surg Treat Res.

[27] Tedesco MM, Amato B, Santoro D (2024). Intraoperative neuromonitoring during carotid endarterectomy: current evidence and future perspectives. J Vasc Surg.

[28] Wang BH, Leung A, Lownie SP (2016). Circle of willis collateral during temporary internal carotid artery Occlusion II: observations from computed tomography angiography. Can J Neurol Sci.

